# Crystal structures of PCNA1 and PCNA2 from *Aeropyrum pernix*: implications for a distorted heterotrimeric sliding clamp

**DOI:** 10.1107/S2053230X26003687

**Published:** 2026-05-19

**Authors:** Tong Wang, Sonoko Ishino, Yoshizumi Ishino, Takuji Oyama

**Affiliations:** a Integrated Graduate School of Medicine, Engineering and Agricultural Sciences University of Yamanashi 4-4-37 Takeda Kofu Yamanashi400-8510 Japan; b Department of Bioscience and Biotechnology, Graduate School of Bioresource and Bioenvironmental Sciences Kyushu University 744 Motooka Nishi-ku Fukuoka819-0395 Japan; c Nagahama Institute of Bio-Science and Technology 1266 Tamura Nagahama Shiga526-0829 Japan; d Institute of Innovative Research, Institute of Science Tokyo, 4259 Nagatsuta-cho, Midori-ku, Yokohama, Kanagawa226-8503, Japan; e Department of Biotechnology, Faculty of Life and Environmental Sciences University of Yamanashi 4-4-37 Takeda Kofu Yamanashi400-8510 Japan; Sungkyunkwan University School of Medicine, Republic of Korea

**Keywords:** *Aeropyrum pernix*, DNA replication, proliferating cell nuclear antigens, heterotrimers, nonproline *cis*-peptides

## Abstract

*Aeropyrum pernix* is a hyperthermophilic archaeon that possesses three proliferating cell nuclear antigen (PCNA) isoforms (ApePCNA1, ApePCNA2 and ApePCNA3) that form a heterotrimeric sliding clamp. To gain more detailed structural insights into this heterotrimeric assembly, we determined the crystal structures of ApePCNA1 and ApePCNA2. ApePCNA1 was crystallized under a new condition, and the 1.60 Å resolution structure revealed a unique non­proline *cis*-peptide bond between Arg187 and Arg188, which was not deeply discussed in a previous report. The structure of ApePCNA2 was determined at 2.17 Å resolution, and it forms a typical homotrimeric ring. In the cubic crystal form, its crystal packing shows an intriguing tetrahedral assembly of four trimers. Modeling the ApePCNA1–ApePCNA2–ApePCNA3 heterotrimer based on these structures suggests that the *cis*-peptide in ApePCNA1 induces significant steric hindrance at the subunit interface, leading to a symmetry-broken or distorted ring conformation rather than the canonical pseudo-threefold-symmetric assembly.

## Abstract 2

Crystal structures of *A. pernix* PCNA1 (1.60 Å resolution) and PCNA2 (2.17 Å resolution) are reported. High-resolution analysis reveals a rare nonproline *cis*-peptide bond in PCNA1 that may cause steric hindrance at the subunit interface, suggesting a distorted or symmetry-broken heterotrimeric PCNA ring.

## 1. Introduction

DNA replication is an essential process that is conserved across all living organisms (O’Donnell *et al.*, 2013 ▸; Bell & Kaguni, 2013 ▸; Ishino *et al.*, 2013 ▸). Efficient DNA replication requires ring-shaped sliding clamps to tether DNA polymerases and other enzymes onto DNA, increasing their processivity. In eukaryotes and archaea, the proliferating cell nuclear antigen (PCNA) homotrimer serves this role, while the β-subunit dimer and the gp45 trimer function in bacteria and phages, respectively (Kong *et al.*, 1992 ▸; Krishna *et al.*, 1994 ▸). DNA-metabolizing enzymes contact PCNA through the PCNA-interacting peptide (PIP) motif or its variants (Warbrick, 1998 ▸; De Biasio & Blanco, 2013 ▸). These clamps are loaded onto DNA by pentameric AAA^+^ clamp loaders (Miyata *et al.*, 2005 ▸; Kelch *et al.*, 2011 ▸; Gaubitz *et al.*, 2022 ▸).

Despite the fundamental differences in cellular organization and their extreme habitats, archaeal DNA-replication systems are remarkably similar to those of eukaryotes (Ishino & Cann, 1998 ▸; Kelman & White, 2005 ▸). Certain Crenarchaeota possess multiple PCNA isoforms (Daimon *et al.*, 2002 ▸; Dionne *et al.*, 2003 ▸). In *Saccharolobus solfataricus* and *Aeropyrum pernix*, three PCNA isoforms assemble into a stable heterotrimeric ring (Pascal *et al.*, 2006 ▸; Williams *et al.*, 2006 ▸; Imamura *et al.*, 2007 ▸; Hlinkova *et al.*, 2008 ▸), which is considered to be evolutionarily related to the eukaryotic Rad9–Rad1–Hus1 heterotrimer for DNA repair (Doré *et al.*, 2009 ▸).

Although detailed intersubunit interactions have been analyzed (Imamura *et al.*, 2007 ▸), structural data for *A. pernix* were restricted to ApePCNA1 (Yamauchi *et al.*, 2024 ▸). Here, we report the crystal structure of ApePCNA1 determined under a new condition and the newly solved ApePCNA2 homotrimer. The ApePCNA1 structure revealed a unique protrusion formed by the nonproline *cis*-peptide bond. Modeling of the ApePCNA1–ApePCNA2–ApePCNA3 heterotrimer based on these structural data suggests that the *cis*-peptide protrusion in ApePCNA1 may induce a distorted or symmetry-broken ring conformation, rather than the canonical threefold-symmetric ring.

## 2. Materials and methods

### 2.1. Macromolecule production

Recombinant ApePCNA1 (UniProt code Q9YFT8, APE_0162) and ApePCNA2 (Q9Y9V7, APE_2182) proteins (Table 1 ▸), without any non-native residues, were expressed and purified following the methods previously described by Daimon *et al.* (2002 ▸). It should be noted that ApePCNA2 was referred to as ApePCNA3 in the previous literature. These proteins were overexpressed in an *Escherichia coli* system. The harvested cells were disrupted, followed by heat treatment, polyethyleneimine treatment in the presence of high-concentration NaCl and ammonium sulfate precipitation to obtain crude extracts. The proteins were then purified using anion-exchange and heparin column chromatography to achieve sufficient purity for crystallization. The purified proteins were concentrated to 20 mg ml^−1^ using Amicon Ultra-4 centrifugal filter units (Merck) for subsequent crystallization experiments.

### 2.2. Crystallization

Crystallization of ApePCNA1 and ApePCNA2 was performed at 298 K using the hanging-drop vapor-diffusion method (Table 2 ▸). We attempted crystallization based on the conditions reported for *Pyrococcus furiosus* PCNA (Matsumiya *et al.*, 2002 ▸), which shares 34% and 29% sequence identity with ApePCNA1 and ApePCNA2, respectively. Single crystals suitable for X-ray diffraction experiments were successfully obtained without extensive screening. Specifically, 1 µl protein solution was mixed with an equal volume of reservoir solution consisting of 0.1 *M* citric acid pH 4.5 and 1.8 *M* ammonium sulfate. The initial drops were equilibrated against 500 µl of the same reservoir solution. Polyhedral crystals with a maximum dimension of approximately 0.2 mm were grown within a week.

### 2.3. Data collection and processing

X-ray diffraction data (Table 3 ▸) were collected on beamline BL38B1 at the SPring-8 synchrotron-radiation facility, Harima, Japan. Prior to data collection, crystals were briefly soaked in a cryoprotectant solution consisting of the reservoir solution supplemented with a final concentration of 20% glycerol. The crystals were then directly mounted and flash-cooled in a stream of cold nitrogen gas at 100 K. Diffraction images were recorded on a Rigaku Jupiter 210 CCD detector. For each dataset, a total of 180 images were collected with an oscillation angle of 1.0° per frame. The ApePCNA1 crystal belonged to the tetragonal space group *P*4_3_2_1_2, with unit-cell parameters *a* = *b* = 68.93, *c* = 120.165 Å, and diffracted X-rays to a resolution of 1.60 Å. The crystal contained one molecule in the asymmetric unit, which led to a Matthews coefficient (*V*_M_) of 2.43 Å^3^ Da^−1^ and a solvent content of 49.4%. The ApePCNA2 crystal belonged to the cubic space group *P*2_1_3 and diffracted to 2.17 Å resolution. The unit-cell parameters for ApePCNA2 were *a* = *b* = *c* = 169.79 Å. The asymmetric unit contained four copies of the protein, with a *V*_M_ of 2.16 Å^3^ Da^−1^ and a solvent content of 43.2%. Data processing was performed using *XDS* (Kabsch, 2010 ▸) and *AIMLESS* (Evans & Murshudov, 2013 ▸).

### 2.4. Structure solution and refinement

The crystal structures of both ApePCNA1 and ApePCNA2 were determined by the molecular-replacement method. Calculations were performed using *Phaser* within the *Phenix* package (Liebschner *et al.*, 2019 ▸). A polyalanine model based on the *P. furiosus* PCNA monomer (PDB entry 1ge8) was used as a search probe. Following the correct placement of the probe in the crystal lattice, initial models including side chains were generated using *phenix.autobuild*. The structures were further improved through cycles of crystallographic refinement using *phenix.refine* and manual model rebuilding with *Coot* (Emsley *et al.*, 2010 ▸) until the refinement converged (Table 4 ▸). Most of the ApePCNA1 structure could be built into the electron-density map, excluding some residues at both termini (Met1–Ser3, Ser9–Asp13 and Gly263). In the case of ApePCNA2, all amino-acid residues in the four molecules of the asymmetric unit were visible in the electron-density map.

## 3. Results and discussion

### 3.1. Molecular arrangement in the crystals

**Figure 1:**
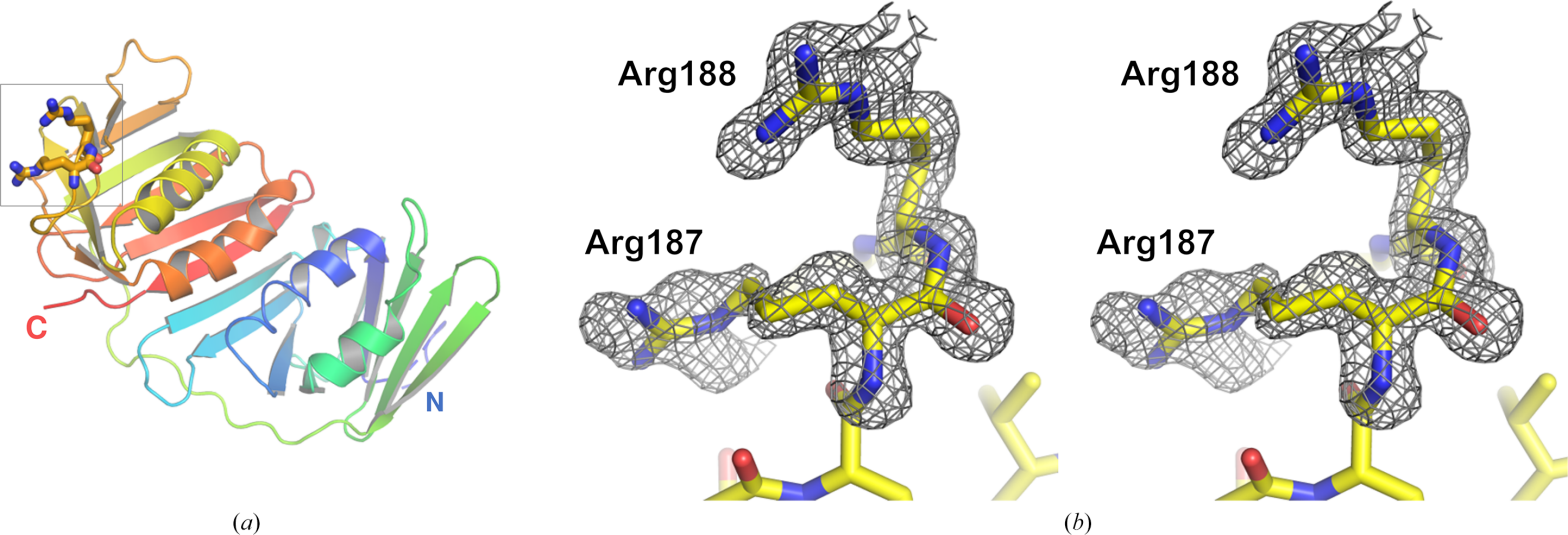
The nonproline *cis-*peptide between Arg187 and Arg188 of ApePCNA1. (*a*) Overall structure of the ApePCNA1 monomer shown as a cartoon model, colored in a rainbow gradient from the N-terminus (blue) to the C-terminus (red). (*b*) Stereo pair of a close-up view showing the *F*_o_ − *F*_c_ omit electron-density map (contoured at 2.5σ) for the unique nonproline *cis*-peptide bond identified between Arg187 and Arg188. All structural figures were prepared using *PyMOL* (Schrödinger).

**Figure 2:**
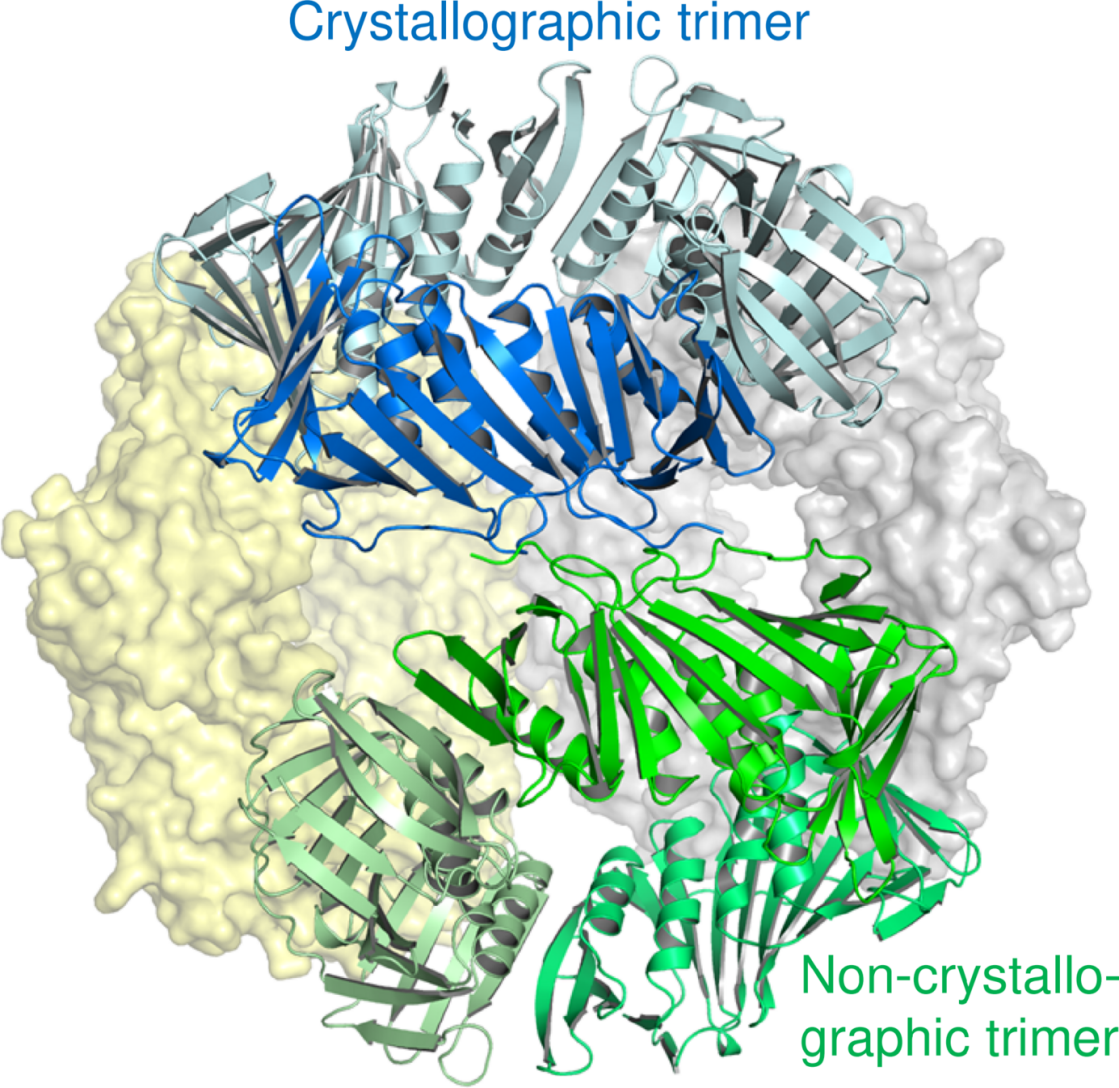
Crystal packing and tetrahedral assembly of ApePCNA2. Among the four molecules in the asymmetric unit, three molecules (chains *A*, *B* and *C*; shown as green cartoons) form a homotrimer via noncrystallographic threefold symmetry. Two additional trimers generated by the crystallo­graphic symmetry are shown as yellow and gray surface models. The remaining molecule (chain *D*; dark blue cartoon) forms another homotrimer with its symmetry mates (light blue cartoons). Consequently, four homotrimers are assembled into a tetrahedral cluster within the crystal lattice.

Despite very similar crystallization conditions and morphologies, ApePCNA1 and ApePCNA2 crystallized in different space groups. The structure of ApePCNA1 was determined at 1.60 Å resolution as a monomer in the same space group and with similar unit-cell parameters as the previously reported structure (PDB entry 6aig; Yamauchi *et al.*, 2024 ▸), with a root-mean-square deviation (r.m.s.d) of 0.37 Å for 252 C^α^ atoms. Notably, we identified a nonproline *cis*-peptide bond between Arg187 and Arg188 in the C-terminal domain (Fig. 1 ▸*b*), a feature that despite being present in PDB entry 6aig was not deeply discussed in the previous study. As discussed below, this unique local conformation likely contributes to the specific assembly characteristics of the *A. pernix* heterotrimer by potentially hindering homotrimerization (Imamura *et al.*, 2007 ▸). For ApePCNA2, four monomers in the asymmetric unit form a noncrystallographic symmetry (NCS)-related trimer (chains *A*–*C*) and a crystallographic trimer (chain *D*), which together assemble into a tetrahedral cluster (Fig. 2 ▸).

### 3.2. Overall structure of the ApePCNA1 and ApePCNA2 subunits

Both ApePCNA1 and ApePCNA2 adopt the canonical PCNA fold, which is characterized by a pseudo-sixfold-symmetric architecture (Figs. 1 ▸ and 2 ▸). Although the inter-domain connecting loop (IDCL) is often disordered in unliganded PCNAs, it was clearly defined in both structures, likely stabilized by crystal packing.

**Figure 3:**
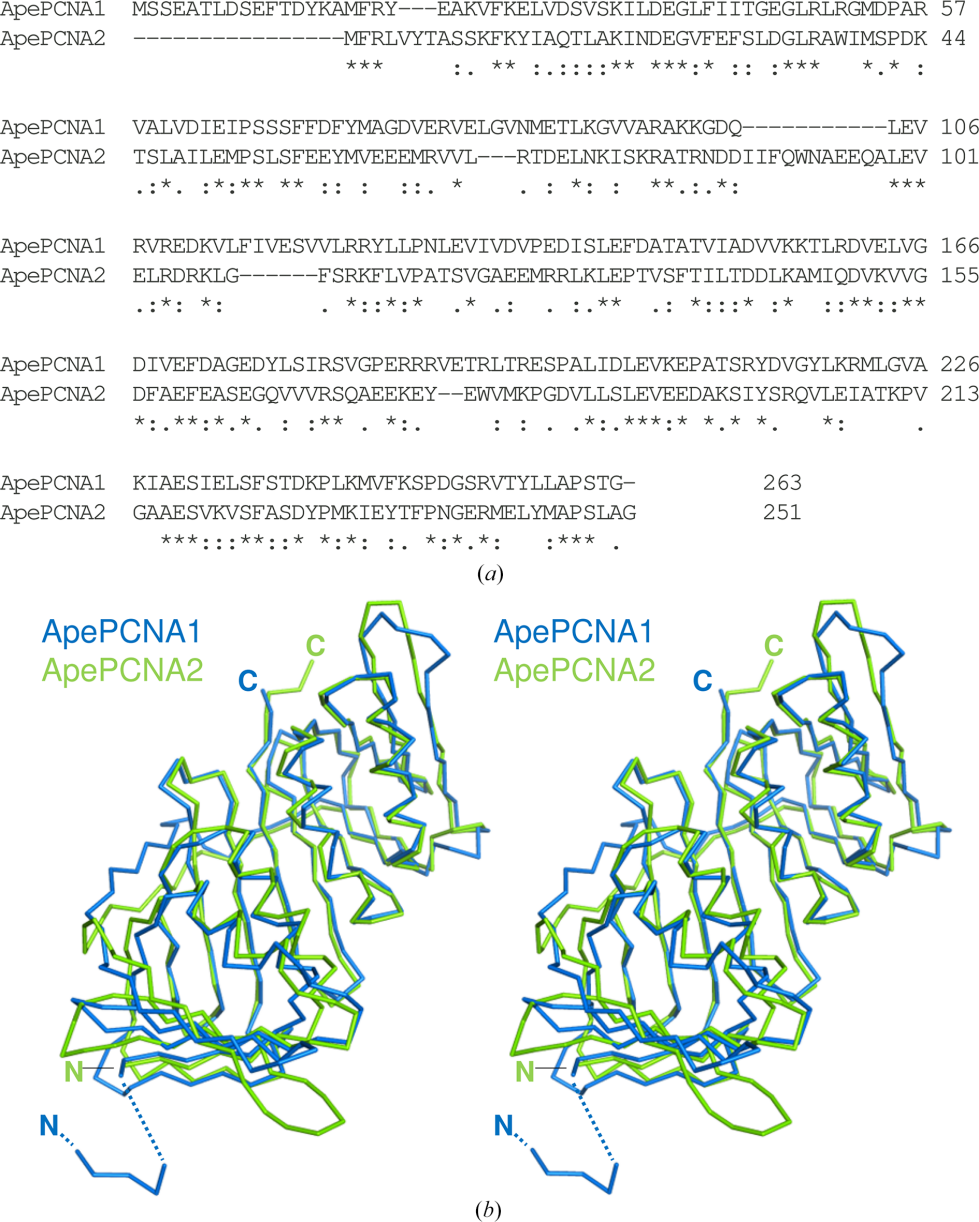
Structural comparison between ApePCNA1 and ApePCNA2. (*a*) Structure-based sequence alignment. Asterisks and colons indicate identical and highly similar residues, respectively. (*b*) Stereoview of the superposed structures. ApePCNA1 and ApePCNA2 are represented as C^α^ traces in sky blue and green, respectively.

ApePCNA1 and ApePCNA2 share 28.7% sequence identity (Daimon *et al.*, 2002 ▸) and exhibit high structural similarity, with an r.m.s.d. of 1.41 Å for 231 C^α^ atoms (Fig. 3 ▸). Similarly, the *AlphaFold*3-predicted ApePCNA3 model aligns closely with ApePCNA2 (r.m.s.d. of 1.37 Å for 239 C^α^ atoms). This structural conservation across the three subunits supports the feasibility of the heterotrimer model described below.

### 3.3. Implications for *A. pernix* PCNA heterotrimer assembly

**Figure 4:**
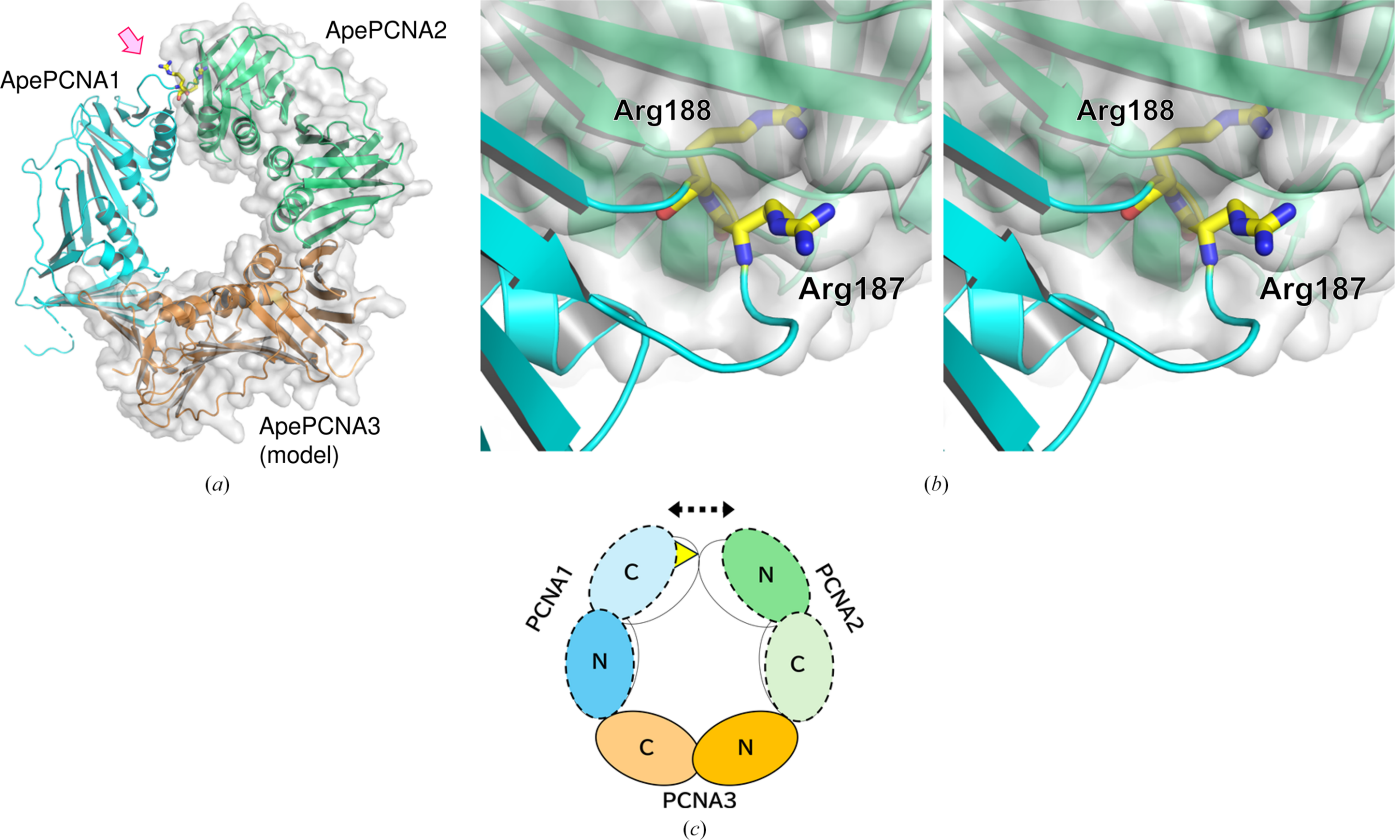
Proposed structural model of the *A. pernix* PCNA heterotrimer. (*a*) Model of the ApePCNA1–ApePCNA2–ApePCNA3 heterotrimer constructed using the ApePCNA2 homotrimer as a template. Subunits are replaced with ApePCNA1 (cyan) and an ApePCNA3 model (orange). The *cis*-peptide is shown as yellow sticks. (*b*) Stereo pair of a close-up view of the clashing region between ApePCNA1 and ApePCNA2, viewed from the direction indicated by the arrow in (*a*). Arg188 causes a steric clash with ApePCNA2. (*c*) Schematic diagram of the predicted ApePCNA heterotrimer. The threefold rotational symmetry is predicted to be broken by the *cis*-peptide protrusion (yellow triangle) of ApePCNA1.

Previous biochemical analysis suggested that the *A. pernix* heterotrimer assembles in the order ApePCNA1–ApePCNA3–ApePCNA2, as ApePCNA3 interacts stably with both ApePCNA1 and ApePCNA2, whereas the interaction between ApePCNA1 and ApePCNA2 is weak (Imamura *et al.*, 2007 ▸). We constructed a heterotrimer model using our ApePCNA2 homotrimer as a template, incorporating the experimental ApePCNA1 structure and an *AlphaFold*3-predicted model (Abramson *et al.*, 2024 ▸) of ApePCNA3, with the *S. solfataricus* PCNA heterotrimer (PDB entry 2ix2) as a reference (Fig. 4 ▸*a*).

This modeling revealed significant steric hindrance at the interface between ApePCNA1 and ApePCNA2 (Fig. 4 ▸*b*), caused by the protruding segment of ApePCNA1 containing the Arg187-Arg188 *cis*-peptide bond. This structural distortion is likely attributed to a unique sequence that forms the *cis*-peptide, rather than an insertion, at the subunit interface of ApePCNA1: a region that, despite its critical role in ring assembly, is surprisingly not conserved among PCNA homologs (Daimon *et al.*, 2002 ▸). *AlphaFold*3 failed to accurately predict this *cis* conformation (data not shown), highlighting the importance of experimental validation for capturing such rare nonproline *cis*-peptides. Since nonproline *cis*-peptide bonds are typically rigid (Jabs *et al.*, 1999 ▸), this segment likely maintains its conformation in solution. Consequently, the *A. pernix* PCNA heterotrimer may adopt a distorted or symmetry-broken ring architecture instead of the canonical pseudo-threefold-symmetric closed ring (Fig. 4 ▸*c*). Experimental determination of the full heterotrimer structure remains a high priority to verify this model.

## Supplementary Material

PDB reference: ApePCNA1, 24ku

PDB reference: ApePCNA2, 24jd

## Figures and Tables

**Table 1:** Macromolecule-production information

Source organism	*Aeropyrum pernix*
Expression vector	pET-21a
Expression host	*Escherichia coli* BL21 (DE3)
Complete amino-acid sequence of the construct produced	
ApePCNA1 (Q9YFT8, APE_0162)	MSSEATLDSEFTDYKAMFRYEAKVFKELVDSVSKILDEGLFIITGEGLRLRGMDPARVALVDIEIPSSSFFDFYMAGDVERVELGVNMETLKGVVARAKKGDQLEVRVREDKVLFIVESVVLRRYLLPNLEVIVDVPEDISLEFDATATVIADVVKKTLRDVELVGDIVEFDAGEDYLSIRSVGPERRRVETRLTRESPALIDLEVKEPATSRYDVGYLKRMLGVAKIAESIELSFSTDKPLKMVFKSPDGSRVTYLLAPSTG
ApePCNA2 (Q9Y9V7, APE_2182)	MFRLVYTASSKFKYIAQTLAKINDEGVFEFSLDGLRAWIMSPDKTSLAILEMPSLSFEEYMVEEEMRVVLRTDELNKISKRATRNDDIIFQWNAEEQALEVELRDRKLGFSRKFLVPATSVGAEEMRRLKLEPTVSFTILTDDLKAMIQDVKVVGDFAEFEASEGQVVVRSQAEEKEYEWVMKPGDVLLSLEVEEDAKSIYSRQVLEIATKPVGAAESVKVSFASDYPMKIEYTFPNGERMELYMAPSLAG

**Table 2:** Crystallization

Method	Hanging-drop vapor diffusion
Plate type	VDX plate
Temperature (K)	293
Protein concentration (mg ml^−1^)	20
Buffer composition of protein solution	50 m*M* Tris–HCl pH 8.0, 0.1 m*M* EDTA, 0.5 m*M* dithiothreitol, 10% glycerol, 250 m*M* (ApePCNA1) or 600 m*M* (ApePCNA2) NaCl
Composition of reservoir solution	0.1 *M* citric acid pH 4.5, 1.8 *M* ammonium sulfate
Volume and ratio of drop	1 µl protein solution and 1 µl reservoir solution
Volume of reservoir (µl)	500

**Table 3:** Data collection and processing Values in parentheses are for the outer shell.

Crystal	ApePCNA1	ApePCNA2
X-ray source	BL38B1, SPring-8	BL38B1, SPring-8
Wavelength (Å)	1.00000	1.00000
Temperature (K)	100	100
Detector	Rigaku Jupiter 210	Rigaku Jupiter 210
Space group	*P*4_3_2_1_2	*P*2_1_3
*a*, *b*, *c* (Å)	68.93, 68.93, 120.17	169.79, 169.79, 169.79
α, β, γ (°)	90, 90, 90	90, 90, 90
Mosaicity (°)	0.30	0.12
Resolution range (Å)	45.3–1.60 (1.63–1.60)	47.1–2.17 (2.25–2.17)
Total No. of reflections	412127 (31722)	1614594 (122163)
No. of unique reflections	38871 (3729)	86020 (8530)
Completeness (%)	99.7 (97.7)	99.9 (100.0)
Multiplicity	10.6 (8.5)	18.8 (14.3)
〈*I*/σ(*I*)〉	20.3 (4.1)	24.82 (4.23)
*R*_meas_	0.076 (0.389)	0.098 (0.686)
*R*_p.i.m._	0.023 (0.131)	0.022 (0.180)
CC_1/2_	0.998 (0.936)	0.999 (0.933)
Overall *B* factor from Wilson plot (Å^2^)	16.49	33.23

**Table 4:** Structure refinement

Crystal	ApePCNA1	ApePCNA2
Resolution range (Å)	45.3–1.60	47.1–2.17
Final *R*_cryst_	0.198	0.205
Final *R*_free_	0.218	0.237
No. of non-H atoms		
Protein	2005	8197
Ligand	0	60 (sulfate ions)
Water	89	168
R.m.s. deviations		
Bond lengths (Å)	0.007	0.005
Angles (°)	0.88	0.73
Average *B* factors (Å^2^)		
Protein	22.86	39.34
Ligand	—	30.84
Water	25.62	35.15
Ramachandran plot		
Most favored (%)	99.2	97.6
Allowed (%)	0.80	2.41
PDB code	24ku	24jd
